# The Effects of Fecal Donors with Different Feeding Patterns on Diarrhea in a Patient Undergoing Hematopoietic Stem Cell Transplantation

**DOI:** 10.1155/2019/4505238

**Published:** 2019-03-31

**Authors:** Jianping Zhang, Guangxu Ren, Minghua Li, Peihua Lu, Suqin Yi

**Affiliations:** ^1^Bone Marrow Transplantation (BMT) Unit, Hebei Yanda Ludaopei Hospital, Sanhe, China; ^2^Institute of Food and Nutrition Development, Ministry of Agriculture, Beijing, China

## Abstract

Almost 90% of patients undergoing hematopoietic stem cell transplantation (HSCT) experience diarrheal episodes, which represent a severe, often life-threatening complication for these patients. Although fecal microbiota transplantation (FMT) represents an alternative treatment option for infection-related diarrhea, the application of FMT in HSCT patients is greatly restricted for safety reasons. Furthermore, the therapeutic outcomes of FMT as a diarrhea treatment are somewhat related to the choice of the FMT donor. Here, we comprehensively profiled the dynamic changes in the intestinal microbiota after FMT from two donors with different feeding patterns and the same severely diarrheal recipient undergoing HSCT via a 45-day clinical observation. Importantly, no adverse events attributed to FMT were observed. The stool volume and frequency of the patient were reduced when we used feces from donor #1 (mixed feeding), but these changes were not observed after FMT from donor #2 (exclusive breastfeeding). Interestingly, no obvious differences in overall diversity (Shannon) or richness (Chao1) between the two donors were observed. Additionally, *Bifidobacterium* accounted for 29.9% and 18.1% of OTUs in the stools of donors #1 and #2, respectively. *Lactobacillus* accounted for 16.3% and 2.9% of the stools of donors #1 and #2, respectively. Furthermore, through longitudinal monitoring of the patient, we identified 6 OTUs that were particularly sensitive to the different FMT complements. Together, we present a case report suggesting that the overall diversity of the intestinal microbiota may not be the only important element in the selection of an effective FMT donor.

## 1. Introduction

Almost 90% of patients undergoing hematopoietic stem cell transplantation (HSCT) [1–4] experience diarrheal episodes during the first 3 months after transplantation [1] and can represent a severe and even life-threatening complication in these HSCT patients [3, 4]. Although the etiology of diarrhea in these patients is often difficult to ascertain, the causes can be roughly divided into infectious events (bacterial and viral gastroenteritis) and noninfectious events [1] (conditioning therapy and acute graft-versus-host disease (GVHD)). Currently, some antidiarrheal agents have been approved to mitigate the symptoms of diarrhea [5], but these drugs do not manage diarrhea for some patients undergoing HSCT in some clinical situations.

The gut microbiome plays important roles in regulating diarrhea-related functions, including as a protective barrier to incoming pathogens by colonization competition [6] and in the development of the immune system. Meanwhile, diarrheal episodes are also associated with the composition of the intestinal microbiota [7–10]. An overall decrease in the phylogenetic diversity of the intestinal microbiota has been observed during diarrheal episodes [7, 8, 11, 12]. For patients undergoing HSCT, the most common causes of diarrhea are intestinal infection and GVHD from day 20 to day 100 posttransplant [13]. Interestingly, loss of microbiota diversity has been associated with increased infections and GVHD [14–18]. Thus, the reversal of this intestinal dysbiosis using fecal microbiota transplantation (FMT) has increasingly been used in clinical practice as treatment for diarrhea caused by *Clostridium difficile* infection that cannot be eliminated with antibiotics alone [19].

Given that FMT carries a potential risk of infection [20, 21], the application of FMT in patients with leukemia, especially in patients undergoing HSCT, has been greatly restricted until recently, when some studies evaluated the safety of FMT in patients with leukemia [22, 23]. In cases where diarrhea could not be mitigated with medicine, FMT has commonly been used to treat HSCT patients. However, we found that the outcomes of FMT were to some extent related to the stool sample donors, which raised the question of how to select appropriate donors. To more precisely address the role of stool donors in the outcomes of diarrhea in HSCT patients, we comprehensively profiled the dynamic changes in the intestinal microbiota between donors with different feeding patterns and the same recipient.

## 2. Case Presentation

Patient #A14, a 56-year-old male subject, was diagnosed with acute myeloid leukemia (AML-M4) in October 2016. A decision was made for haploidentical HSCT with his son as the donor on 28 March 2017. He received grafts from 5/10 HLA-matched peripheral blood stem cells (PBSCs) and bone marrow (BM) stem cells. The conditioning regimen was modified BUCY (busulfan: 3.2 mg/kg, iv days −9 to −6; cyclophosphamide: 1.8 g/m^2^, days −5 to −4); GVHD prophylaxis consisted of antithymocyte globulin (ATG), cyclosporin A (CSA), mycophenolate mofetil (MMF), and short-term methotrexate (sMTX). ATG (thymoglobulin, rabbit; Genzyme Europe B.V., Naarden, the Netherlands) was given at a dose of 10 mg/kg from days −5 to −2. CsA (3 mg/kg, iv every 12 h) was administered starting on day −10, and the trough concentration was adjusted to 150–300 ng/ml. MMF was administered orally starting on day −10 (0.5 g, every 12 h) and was withdrawn on day +45 for haploidentical donor (HID) HSCT. sMTX was administered intravenously at 15 mg/m^2^ on day +1 and 10 mg/m^2^ on days +3, +6, and +11. Neutrophil and platelet engraftment occurred on days +15 and +23, respectively.

One month later, on day +27, the patient developed abdominal tenderness and diarrhea. In addition to abdominal pain, diarrhea, and fever, patient #A14 had no GVHD-related symptoms and no impaired liver function (alanine aminotransferase, aspartate aminotransferase, total bilirubin, and direct bilirubin were normal). Because of insufficient evidence of GVHD, neither enteroscopy (sigmoidoscopy and colonoscopy) nor glucocorticoid therapy was performed for this patient. Virological tests showed that cytomegalovirus (CMV) DNA and Epstein–Barr virus (EBV) DNA were not present in the blood. Antiviral therapy was given acyclovir (0.4 Po Bid) to prevent herpes virus infection. Although the patient's temperature returned to normal and abdominal pain relieved after anti-infection treatment, the diarrhea did not alleviate. Stool screening showed no *Clostridium difficile* infection. Fecal neutrophils were not found in stained smears of diarrheal stools. We did not observe obvious improvement after we treated the patient with Smecta combined with sulperazone for three days. The ratio of cocci to bacilli was 9 : 1 by stool smear. One week after cessation of medical treatment, we attempted to use FMT to treat the diarrhea.

## 3. Methods

### 3.1. Study Design

**Figure 1:**
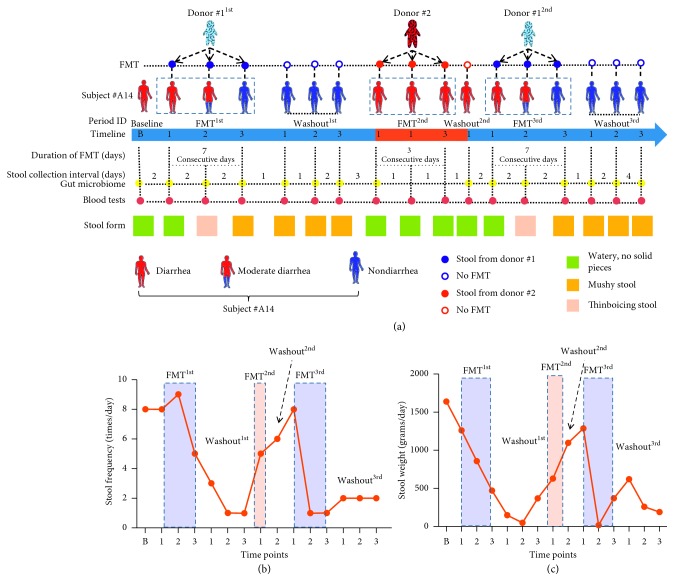
Differences in the effects of stools from donors with two feeding patterns on outcomes of diarrhea treatment via FMT in the same recipient. (a) Illustration of this study design, which was a longitudinal observation comparing the effects of stool samples from two donors with different feeding patterns (donor #1 was mixed fed, and donor #2 was exclusively breastfed) on the therapeutic outcomes of diarrhea via FMT in the same recipient (subject #A14). Thinboicing stool is the transitional form between watery stool and musy stool. The stool frequency (b) and weight (c) of the recipient were continuously recorded during the entire study.

The first stage of FMT (FMT^1st^) was performed on day 2 after cessation of diarrhea treatment (Figure 1(a)). Fecal samples were obtained from a 1-year-old female infant (donor #1) who had a mixed feeding pattern (formula feeding and complementary food). Doses of 60 mL of fecal suspension were administered for 7 consecutive days via a nasoduodenal tube. Then, we suspended the FMT operation and continually observed the dynamic changes in the intestinal microbiota. However, one week after cessation of FMT, diarrhea recurred in this patient. Unfortunately, stool samples from donor #1 were not available on that occasion. Thus, we used fecal samples from another 1-year-old male infant (donor #2) who had an exclusive breastfeeding pattern. After 3 consecutive days of FMT^2nd^, we did not observe any improvement in the diarrhea, and we terminated FMT on day 4 (Figure 1(a)). Two days later, we obtained additional stool samples from donor #1 and conducted the third FMT (FMT^3rd^) for another 7 consecutive days. Afterward, we similarly suspended FMT and continually observed the dynamic changes in the intestinal microbiota for one week. A standardized protocol for donor screening and FMT administration developed by infectious disease and BM transplant specialists was approved by the hospital quality and safety officer. This study was approved by the hospital ethics committee. Informed consent was obtained from both the parents of the stool donors and the FMT recipient. The study was conducted in accordance with the Declaration of Helsinki.

### 3.2. Donor Characteristics

Donor #1, a 1-year-old female infant, was born term at gestational age 39 weeks via C-section, and her birth weight was 2700 g. Donor #2 was a 1-year-old male infant who was delivered though C-section at gestational age 42 weeks, and his birth weight was 3900 g. Donor #1 received mixed feeding from the fifth month after birth to the start of this study. The mixed-fed donor was predominantly fed complementary food and received formula feedings twice a day (the total dietary nutrient intake is shown in Table 1). Donor #2 was exclusively breastfed from birth to the start of this study. Both donors were healthy, without any gastrointestinal pathology or history of antibiotic usage. All donors tested negative for anti-HIV (human immunodeficiency virus), anti-HTLV-1 (human T-lymphotropic virus type I), and antihepatitis A, B, and C. No symptoms of infectious gastroenteritis, such as fever, abdominal pain, nausea, vomiting, or diarrhea, were present on the day of FMT.

### 3.3. FMT Preparation

Fresh stool samples from healthy donors were collected using sterile bags and immediately transported to the laboratory within 5 h of collection. For each specimen, 5 g of the stool sample was stored in a liquid nitrogen tank for future microbiota analysis. The remaining sample was used for FMT according to the protocol described by Lee et al. [24] with minor modification. Briefly, approximately 60 g of the stool sample was diluted with 60 mL of physiological saline solution and emulsified using a sterile glass rod. After filtering out the solids via a medical gauze, 50 mL of the liquid slurry was aspirated into 60 mL syringes and then used to perform FMT within 6 h.

### 3.4. Sequencing Methods

DNA was extracted from thawed stool samples using the E.Z.N.A. Stool DNA kit (Omega Bio-Tek, Norcross, GA, US) according to the manufacturer's instructions. The quality of the DNA was evaluated by 2% agarose gel electrophoresis and spectrophotometry. The V3-V4 region of the 16S rRNA gene was amplified using FastPfu DNA Polymerase (TransStart^TM^, TransGen Biotech). The primers were used as follows: 338F, 5′-ACTCCTACGGGAGGCAGCAG-3' and 806R, 5′-GGACTACHVGGGTWTCTAAT-3'. The PCR cycling conditions were as follows: 95°C for 5 min; 28 cycles of 45 s at 95°C, 50 s at 55°C, 45 s at 72°C, and a final extension at 72°C for 10 min. Purified amplicons were pooled in equimolar amounts and paired-end sequenced (2 × 300) on an Illumina MiSeq platform according to the standard protocols. The sequence data were compiled and filtered for quality. The unique sequence set was classified into operational taxonomic units (OTUs) under a threshold of 97% identity using UCLUST. Chimeric sequences were identified and removed using USEARCH (version 8.0.1623). The taxonomy of each 16S rRNA gene sequence was analyzed by UCLUST against the Silva 119 16S rRNA database using a confidence threshold of 90%. Taxonomic assignments of each OTU were made by similarity using the GLSEARCH program. Alpha diversity values were calculated using Faith's phylogenetic diversity [25].

### 3.5. Statistical Analysis

Data were analyzed using a combination of the software programs R and Mothur. Heat maps, principal coordinates analysis (PCoA), and bar plots were created in R. The nonparametric Kruskal–Wallis test was performed using GraphPad Prism 4.0 software (GraphPad Inc., San Diego, California, USA). *P* values less than 0.05 were considered significant.

## 4. Case Outcomes

### 4.1. Effects of Different Infants' Feces on Diarrhea in a Leukemia Patient

Patient #A14 developed sustained abdominal tenderness and diarrhea after allo-HSCT. Given that both Smecta and sulperazone had no effect on diarrhea status in this case, FMT was recommended and then accepted by the subject. Diarrhea status was routinely monitored via frequency, weight, and shape of bowel movements each day during the entire study period (Figures 1(a)–1(c)). During the B phase (the baseline time point: on day 2 after cessation of medical treatment for diarrhea), patient #A14 had 8 liquid bowel movements a day, and the wet weight of the stool reached 1640 g in 24 h (Figures 1(b) and 1(c)). During the first stage of FMT (FMT^1st^), in which stool samples were obtained from donor #1, although the frequency of liquid stools was increased at the beginning of FMT^1st^, the number of bowel movements began to decline significantly from the third sample collection point (3FMT^1st^) onward. Meanwhile, the patient stool mass was dramatically decreased from 1260 g (1FMT^1st^) to 470 g (3FMT^1st^). During the 7-day follow-up after cessation of the first FMT, the shape of the feces was obviously improved (Figure 1(a)), and the frequency of bowel movements gradually decreased from 5 times at 3FMT^1st^ to once at the third sampling time point of the first washout phase (3Washout^1st^, Figure 1(b)). However, after the 3Washout^1st^ phase, the patient developed serious diarrhea again. Next, we used donor #2's stool to perform FMT^2nd^, but we did not observe any improvement during the 3-day FMT process. Following a 3-day washout period (Washout^2nd^), we returned to stool samples from donor #1 to conduct the third FMT (FMT^3rd^). Similarly, during the 7-day FMT period, we found significant improvements in both the shape and frequency of the patient's stool (Figures 1(a) and 1(b)). After cessation of FMT, we continuously observed for another seven days and found no recurrence of diarrhea in patient #A14.

### 4.2. Patterns of Diversity and Richness in the Intestinal Microbiome during FMT

**Figure 2:**
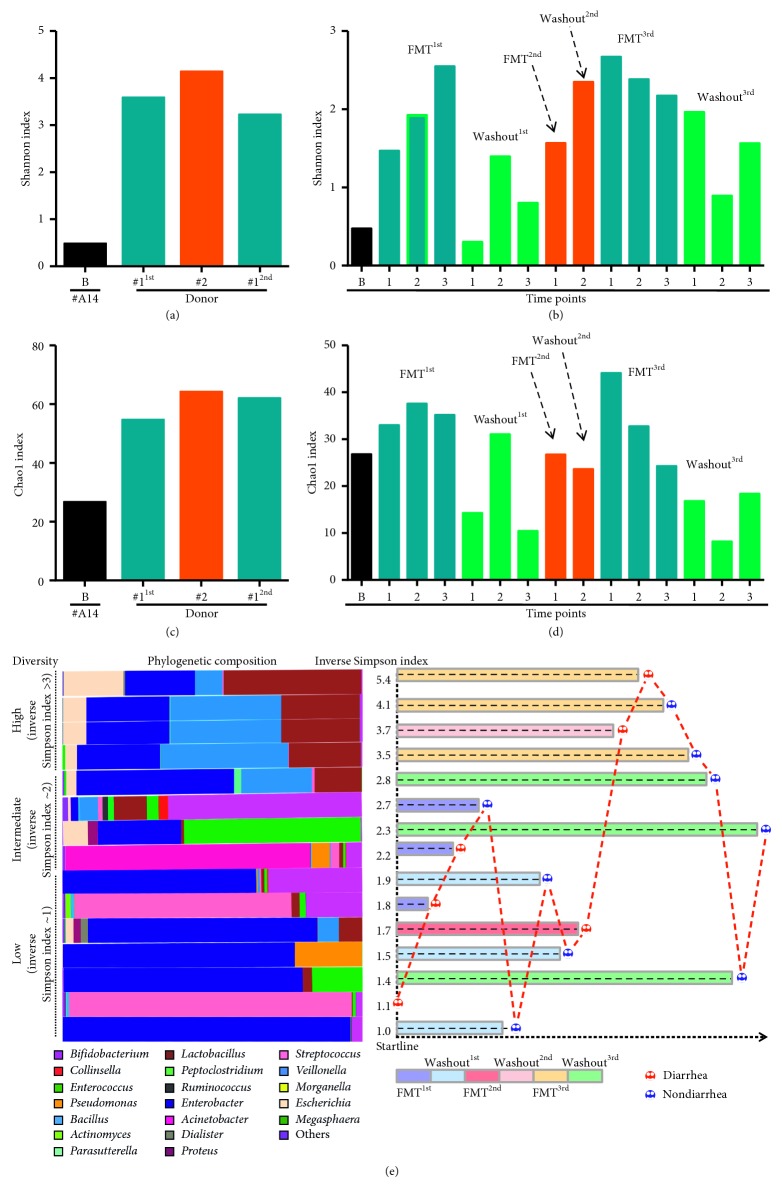
Dynamic changes in microbial diversity and richness during the entire FMT process. Microbial diversity was measured by the Shannon index at baseline (a) and the indicated time points (b) for both the FMT donors and the FMT recipient. Microbial richness was evaluated via the Chao1 index at baseline (c) and the indicated time points (d) during the entire FMT process. Recipient specimen collection points were rearranged according to the level of microbial diversity, and diarrhea status is labeled on the right. (e) The horizontal stacked bars on the left side represent the phylogenetic composition of the recipient at each sample collection time point. The hierarchy of microbial diversity was determined by the inverse Simpson index. Time bars are shown in the columns; the length of the time bar depends on the sequence of specimen collection.

To assess the dynamic changes in the gut microbiome during FMT, we sequenced DNA from stool samples collected at the baseline (B point) and the indicated time points (Figure 1(a)) with 16S rRNA gene sequencing. During allo-HSCT, patients normally undergo dramatic alterations of their intestinal microbiota [26]. In this study, we also found that the overall bacterial diversity (Shannon a-diversity = 0.47) and richness (Chao1 index = 26.75) of patient #A14 were significantly reduced at the beginning of FMT (Figures 2(a) and 2(c)). Although we observed that stool samples from donor #1 were more effective for the treatment of diarrhea than those from donor #2, there were no obvious differences in diversity (Shannon) or richness (Chao1) between the two donors (Figures 2(a) and 2(c)). During the entire FMT process, we observed significantly increased bacterial diversity in the patient after the introduction of stool from either donor (FMT^1st^, FMT^2nd^, and FMT^3rd^; Figures 2(b) and 2(d)). Notably, most bacteria tended not to be retained in the patient's intestine after the cessation of a 1-week-long FMT period, regardless of which stool we used (Chao1 index at Washout^1st^, Washout^2nd^, and Washout^3rd^ time points; Figures 2(b) and 2(d)). We next asked whether the diversity of bacteria affected the occurrence of diarrhea. To this end, we classified the overall samples we sequenced into three different levels based on the inverse Simpson index and correlated diarrhea status with bacterial diversity (Figure 2(e)). In samples with high diversity (inverse Simpson index > 3), diarrhea was observed in 40% (2/5) of samples. Interestingly, these samples shared the same dominant genera, including *Lactobacillus*, *Veillonella*, and *Enterobacter*. Only one sample of the 4 samples with the middle level of diversity presented signs of diarrhea (inverse Simpson index 2.0–2.9). However, the dominant genera were different among these samples. In samples with low diversity (inverse Simpson index < 2), signs of diarrhea were found in 42.8% (3/7) of samples. *Enterobacter* was the dominant genus in 71.4% (5/7) of the samples.

### 4.3. Composition of the Intestinal Microbiome during FMT

**Figure 3:**
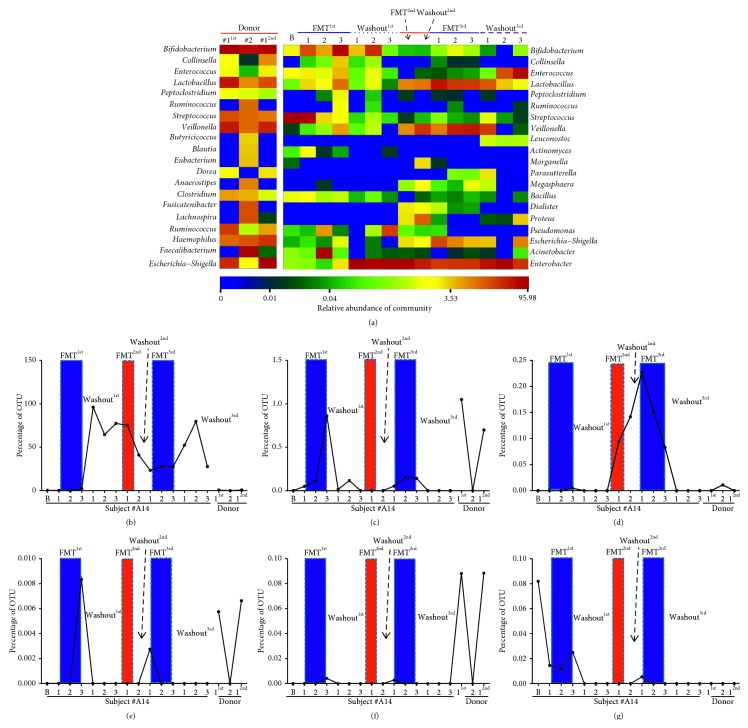
Dynamic changes in composition in the intestinal microbiota. (a) OTU heatmap of all the samples from both donors and the recipient during the entire FMT process. The thermal analysis shows the top 20 OTUs based on their relative abundances. The names on the left are the species annotations for each outlined OTU, and the color value of each square in each row indicates the relative abundance of that OTU. Dynamic changes in 6 OTUs were selected (b–g) from the 114 identified OTUs of patient #A19 based on their sensitivity to FMT.

Based on OTU abundance, we outlined the top 20 genera for each sample (Figure 3(a)). During the baseline measurement, we found that the core microbiome of the patient was *Streptococcus*, which accounted for 94.3% of the sampled OTUs, while *Bifidobacterium* and *Enterococcus* accounted for only 2% and 1.2%, respectively. For the donors, we observed that *Bifidobacterium* (29.8%) and *Faecalibacterium* (28.8%) were the dominant genera in donors #1 and #2, respectively. Other highly represented genera in both donors included *Lactobacillus* (donor #1 = 16.3%, donor #2 = 2.9%), *Veillonella* (donor #1 = 15%, donor #2 = 4.6%), *Streptococcus* (donor #1 = 6.6%, donor #2 = 4.8%), *Haemophilus* (donor #1 = 4.6%, donor #2 = 6.2%), and *Escherichia-Shigella* (donor #1 = 11.3%, donor #2 = 0.6%). Notably, similar compositions were observed in the samples from donor #1 in both FMT^1st^ and FMT^2nd^ procedures (Figure 3(a)). Sequences were assigned to 114 OTUs in patient #A14, and we followed the dynamic changes in each OTU during the entire FMT process. When the patient received the first FMT from donor #1, he showed favorable recovery from diarrhea, and the microbiota composition was dominated by *Streptococcus* (72.2%) and *Bifidobacterium* (18.6%). During the second FMT from donor #2, the microbiota mainly comprised *Enterobacter* (76.5%), *Lactobacillus* (7.8%), and *Veillonella* (7.0%). When the patient received the final FMT from the second donor #1 sample, *Lactobacillus* (23.8%), *Escherichia-Shigella* (19.9%), and *Enterobacter* (23.3%) were the core OTUs of the microbiome. Meanwhile, we identified 6 OTUs that were more sensitive to changes during FMT (OTU_01: *Enterobacter* spp., OTU_29: *Lactobacillus* spp., OTU_52: *Campylobacter* spp., OTU_104: *Ezakiella* spp., OTU_80: *Edwardsiella* spp., and OTU_86: *Luteibacter* spp.) among the 114 recognized OTUs (Figures 3(b)–3(g)).

### 4.4. Comparison of Diarrhea and Nondiarrhea Stages

**Figure 4:**
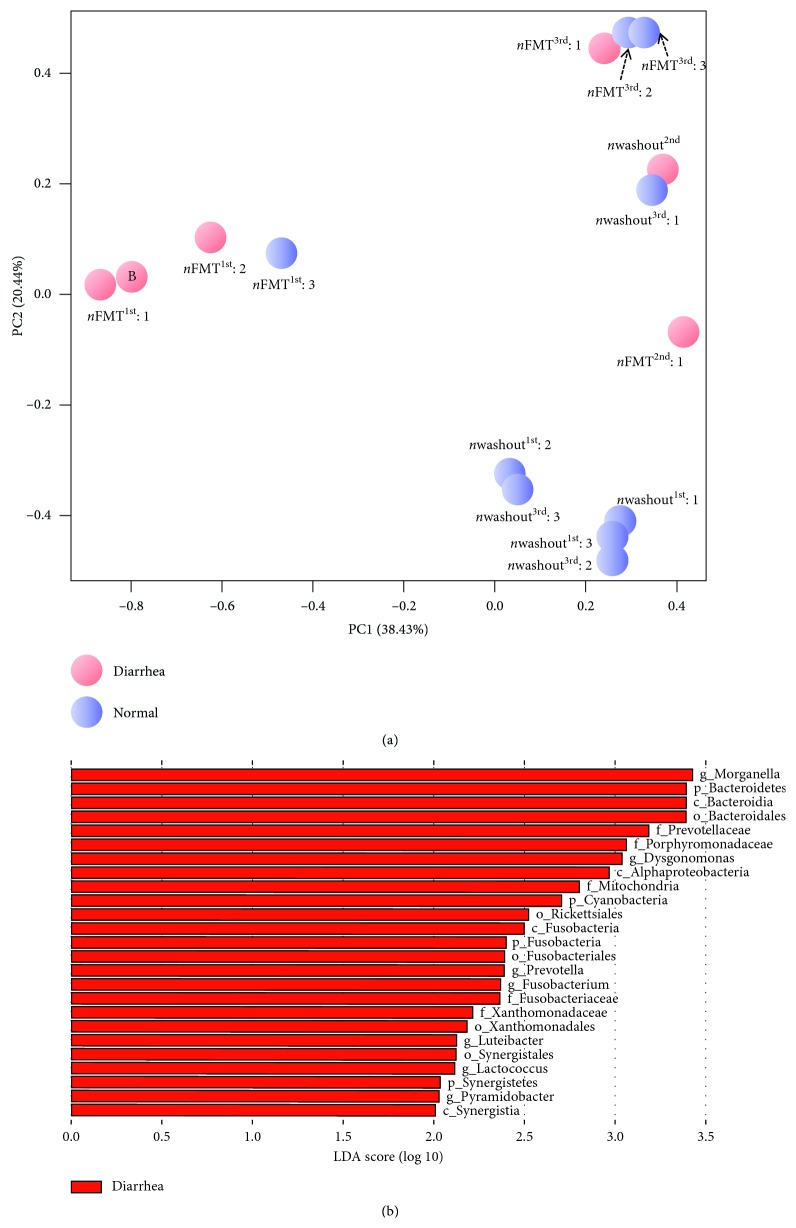
The differences in microbiota species between diarrhea and nondiarrhea stages. (a) Principal coordinates analysis of microbiota species composition of all samples during the whole study. Principal coordinate 1 (PC1, *x*-axis) explains 38.43% of the variance, and principal coordinate 2 (PC2, *y*-axis) explains 20.44% of the variation in Bray–Curtis dissimilarity between samples grouped by diarrhea status (pink circle = diarrhea, blue circle = normal). (b) LDA coupled with effect size measurements identifies the most differentially abundant taxa between diarrhea and normal status. Diarrhea-enriched taxa are indicated with a positive LDA score (red). No obvious differentially abundant taxa were observed in samples with normal status (not shown). Only taxa meeting an LDA significance threshold of >2 are shown.

A PCoA of unweighted UniFrac values identified significant differences in the patient stool microbiome according to whether diarrhea was occurring or not. The samples from time points with normal stool status (Washout^1st^: 1, 2, and 3; Washout^3rd^: 2 and 3) segregated from the other samples (Figure 4(a)). We noted that these samples from the same patient clustered closely and continuously together. To further understand the differences in the microbial communities between diarrhea and nondiarrhea (normal) stages, we also performed linear discriminant analysis effect size (LEfSe) analyses with a logarithmic (LDA) value of 2.0. A total of 7 genera (*Morganella*, *Dysgonomonas*, *Prevotella*, *Fusobacterium*, *Luteibacter*, *Lactococcus*, and *Pyramidobacter*; Figure 4(b)) were found to be significantly different between samples with signs of diarrhea and normal samples. However, no apparent biomarker genera were identified in the nondiarrhea stage.

### 4.5. Dietary Patterns of Subjects

Given that diet has an important influence on the composition of the human intestinal microbiota [27], we closely monitored the daily dietary intake of all subjects using a 24-h dietary recall questionnaire that was used to calculate the total dietary nutrient intake. We noted no significant differences between donor #1, who received mixed feeding (formula feeding + complementary food), and donor #2, who received exclusive breastfeeding, in total dietary nutrient intake, including calories (donor #1 vs. #2 : 65.6 ± 4.2 kcal/kg/day vs. 100 ± 1.2 kcal/kg/day), protein (donor #1 vs. #2 : 2.3 ± 0.7 g/kg/day vs. 2 ± 0.5 g/kg/day), and fat (donor #1 vs. #2 : 1.2 ± 0.8 g/kg/day vs. 5.3 ± 1.5 g/kg/day) (Table 1). For patient #A14, we found that rice paste (50 g rice/meal) and egg (1 egg/meal) were the main daily foods during the whole study (Table 1).

## 5. Discussion

Diarrhea induced by unclear factors including infectious events and noninfectious events in patients undergoing HSCT is common [28], frequently causes notable morbidity and mortality and is often persistent, profuse, and incompletely responsive to symptomatic management [3, 28]. Given that the mechanisms inducing diarrhea in these cases remain poorly understood, its treatment can be challenging, especially for patients undergoing HSCT. The introduction of intestinal microbiota from a healthy donor to restore the indigenous gut microbiota, commonly known as FMT, has been successfully used to treat recurrent *C. difficile*-associated diarrhea when standard therapy fails [29, 30]. Because FMT carries a potential risk of infectious disease transmission [21], the application of FMT has been used with caution in HSCT patients, who have a severely immunosuppressed status.

Recently, Bilinski et al. found that FMT in patients with blood disorders promotes eradication of antibiotic-resistant bacteria (ARB) from the gastrointestinal tract [31]. A pilot study administering FMT by oral capsules early after bone marrow transplantation (BMT) showed that third-party FMT could restore intestinal microbiome diversity after allo-HSCT [32]. Furthermore, a multicenter retrospective series investigated the use of FMT in immunocompromised (IC) patients with *Clostridium difficile* infection [33]. This study found that there were no related infectious complications in these IC patients. Therefore, FMT can still be applied to HSCT patients on the premise of strict control of the FMT operation process. In the clinic, we found that the residues of some irritating foods can induce GVHD in patients undergoing HSCT (data not shown). As we know, adults have a more complex diet than that of 1-year-old infants. Thus, for safety reasons, we used feces from infants instead of adults to perform FMT. Infants 1 year of age have more *Bifidobacteria* than do individuals of other age groups [34]. Several trials have suggested that Bifidobacterial preparations have efficacy in the prevention and treatment of pediatric antibiotic-associated diarrhea [35]. Importantly, the infant intestinal microbiome is significantly associated with the feeding method [36]. This raises the question of what types of infant donors are better suited for FMT. Our study attempts to solve this problem by using feces from two infants with different dietary patterns.

The community of microorganisms within the human gut is critical to health and functions with a level of complexity comparable to that of an organ system [37]. Therefore, dysbiosis of the intestinal microbiome has been implicated in a number of diarrheal diseases [38, 39]. Alterations in the intestinal microbial community have been observed in recurrent *C. difficile*-associated diarrhea [39]. Although *C. difficile* infection was not found in this HSCT patient, the diversity and fitness levels of the fecal microbiome were extremely low at baseline compared with those of a healthy person. Thus, we tried to use feces from healthy donors to rebuild the intestinal microecology of this severe diarrheal patient undergoing HSCT. As we assumed, the diversity and richness of the patient's microbiome were significantly improved after FMT, regardless of the donor that we used. In accordance with previous reports [40, 41], we also found that the bacteria of the two infant donors were largely restricted to a small subset of the bacterial world, namely, *Bifidobacteria*, *Streptococci*, and *Lactobacillus* spp. Increasing evidence has now demonstrated that some microbial species (e.g., *Lactobacilli* and *Streptococcus*) can restore the disrupted gut epithelium that causes the diarrhea commonly experienced by HSCT patients through upregulation or phosphorylation of tight junction proteins [42–44]. Despite this, there was an obvious difference in diarrhea treatment outcomes between these two FMT donors. Through a small FMT substitution, we observed that stool volume and frequency were reduced when we used feces from donor #1, but these effects were not observed after the transplant from donor #2. Our and previous studies have shown that not all FMT is fully effective. Thus, choosing an appropriate FMT donor is particularly important for clinicians when they use FMT to treat diarrhea. However, the mechanisms underlying the beneficial role of FMT in diarrhea have been poorly understood until now, especially for noninfectious diarrhea.

To more precisely address the roles of stool donors in the outcomes of diarrhea in HSCT patients, we comprehensively profiled the dietary patterns of the donors and recipient and the dynamic changes in the intestinal microbiota using a small FMT substitution. Given that HSCT is an intensely immunosuppressive procedure, for safety reasons, we chose two 1-year-old infants whose feeding patterns were relatively simple as FMT donors. During the first year of life, the human infant intestinal microbiome undergoes rapid maturation [45] and is similar to that of an adult in some regions [46]. Importantly, feeding pattern has been shown to influence the structure and function of the intestinal microbiota in infants [45]. Consistent with a previous study [47], we identified Bifidobacteriaceae and *Faecalibacterium* as the dominant taxa in the stool of the exclusively breastfed donor #2 (Figure 3(a)). In contrast, the introduction of solid foods has been associated with increased populations of *Bacteroides* [48] and decreased populations of *Bifidobacteria*, *Enterobacteria*, and some *Clostridium* species [49]. Although the predominant bacterial families in mixed-fed infants have not been well characterized [45], here we found that the microbiome of the mixed-fed infant (donor #1) was more diverse, with greater proportions of *Bifidobacterium*, *Lactobacillus*, *Streptococcus*, *Veillonella*, *Clostridium*, *Ruminococcus*, *Haemophilus*, and *Escherichia-Shigella* (Figure 3(a)). The mixed feeding pattern exposes infants to nondigestible plant carbohydrates, animal protein, and fats, providing new substrates for the survival and dominance of bacterial species not supported by breast milk [49]. The different microbial configurations were also associated with functional shifts, as the increased abundance of *Bacteroides* promoted by the introduction of solid foods has been correlated with increased short-chain fatty acid levels [48].

Antibiotic-associated diarrhea can be reduced to 8% in patients with the administration of *Lactobacillus casei* ssp. *rhamnosus* (*Lactobacillus GG*) [50]. Animal experiments have also confirmed that prophylactic administration of VSL #3 (a mixture of four species of *Lactobacilli*, three species of *Bifidobacteria*, and *Saccharomyces boulardii*) can prevent irinotecan-related diarrhea, and *Lactobacillus GG* can reduce the frequency of severe diarrhea caused by 5FU-based chemotherapy [51]. In our case, *Bifidobacterium* accounted for 29.9% of the OTUs in the stool of donor #1 and 18.1% in the stool of donor #2 (Figure 3(a)); meanwhile, *Lactobacillus* accounted for 16.3% and 2.9% in the stools of donor #1 and donor #2, respectively (Figure 3(a)). Interestingly, the microbiota of donor #1 was characterized as having greater proportions of *Escherichia-Shigella* (11.3%) than that of donor #2 (0.6%). Furthermore, through longitudinal monitoring of the patient, we outlined 6 OTUs that were more sensitive to changes caused by FMT (OTU_01: *Enterobacter* spp., OTU_29: *Lactobacillus* spp., OTU_52: *Campylobacter* spp., OTU_104: *Ezakiella* spp., OTU_80: *Edwardsiella* spp., and OTU_86: *Luteibacter* spp.) among the 114 recognized OTUs (Figures 3(b)–3(g)). According to the outcomes of diarrhea, *Lactobacillus* spp. (OTU_29) might have been one of the possible factors, reducing the incidence of diarrhea when we used stool from donor #1.

Overall, we report the confirmation of the safety of FMT for treating an HSCT patient with diarrhea. FMT offers promise as a potential treatment option for noninfectious diarrhea after HSCT. Importantly, the therapeutic outcomes of diarrhea were somewhat related to the choice of FMT donors. Here, we found that 1-year-old infants can serve as safe FMT donors for treating diarrhea in HSCT patients. Furthermore, the overall diversity of the intestinal microbiota may not be the only important element in the selection of an effective FMT donor. The richness of probiotics, including *Lactobacillus*, *Bifidobacteria*, and *Saccharomyces boulardii*, might be the key factor in choosing the right FMT donor when clinicians treat noninfectious diarrhea. Because this was a small FMT substitution, we are expanding this work by building a larger cohort and using a machine learning method to predict the effects of FMT on diarrhea treatment in leukemia patients by analyzing the intestinal microbiota of FMT donors and recipients.

## Figures and Tables

**Table 1:** Characteristics and dietary patterns.

Subjects	Items						
	Age (years)	Gender	Body weight (kg)	Dietary pattern	Total dietary nutrient intake (mean ± SD)		
					Calories (kcal/kg/day)	Protein (g/kg/day)	Fat (g/kg/day)
A14 (recipient)	56	Male	58	Rice paste (50 g rice/meal) and egg (1/meal)	11.5 ± 2.3^*∗*^	0.38 ± 0.3	0.4 ± 0.2^※^
Donor #1	1	Female	10	Mixed fed (formula feeding + complementary food^※^)	65.6 ± 4.2	2.3 ± 0.7	1.2 ± 0.8
Donor #2	1	Male	11	Exclusively breastfeeding	100 ± 1.2	2 ± 0.5	5.3 ± 1.5

*Note*. The Kruskal–Wallis test was used to calculate the significant differences among A14, donor #1 and donor #2. ^*∗*^A14 vs. donor #1, *p* > 0.05; donor #1 vs. donor #2, *p* > 0.05; A14 vs. donor #2, *p* < 0.05. ^※^Complementary food includes rice paste, formula and yolk.

## Data Availability

The datasets generated during and/or analyzed during the current study are available in the NCBI sequence Read Archive under BioProject PRJNA437812 (SRA project: SRP135146, http://www.ncbi.nlm.nih.gov/sra).

## Abbreviations

HSCT: Hematopoietic stem cell transplantation
FMT: Fecal microbiota transplant
GVHD: Graft-versus-host disease
PCoA: Principal coordinates analysis
OTUs: Operational taxonomic units
LEfSe: Linear discriminant analysis effect size
LDA: Linear discriminant analysis
FMT^1st^: The first FMT
FMT^2nd^: The second FMT
FMT^3rd^: The third FMT
Washout^1st^: The first washout
Washout^2nd^: The second washout
Washout^3rd^: The third washout
*n*FMT^1st^, *n*FMT^2nd^, and *n*FMT^3rd^: *n* is the time of sample collection during the FMT phase
*n*washout^1st^, *n*washout^2nd^, and *n*washout^3rd^: *n* is the time of sample collection during the washout phase.

## References

[1] van Kraaij M. G., Dekker A. W. (2000). Infectious gastro-enteritis: an uncommon cause of diarrhoea in adult allogeneic and autologous stem cell transplant recipients. *Bone Marrow Transplantation*.

[2] Bobak D., Arfons L. M., Creger R. J., Lazarus H. M. (2008). Clostridium difficile-associated disease in human stem cell transplant recipients: coming epidemic or false alarm?. *Bone Marrow Transplantation*.

[3] Avery R., Pohlman B., Adal K. (2000). High prevalence of diarrhea but infrequency of documented Clostridium difficile in autologous peripheral blood progenitor cell transplant recipients. *Bone Marrow Transplantation*.

[4] Tomblyn M., Gordon L., Singhal S. (2002). Rarity of toxigenic Clostridium difficile infections after hematopoietic stem cell transplantation: implications for symptomatic management of diarrhea. *Bone Marrow Transplantation*.

[5] Schiller L. R. (2017). Antidiarrheal drug therapy. *Current Gastroenterology Reports*.

[6] Buccigrossi V., Nicastro E., Guarino A. (2013). Functions of intestinal microflora in children. *Current Opinion in Gastroenterology*.

[7] Monira S., Shabnam S. A., Alam N. H., Endtz H. P., Cravioto A., Alam M. (2012). 16S rRNA gene-targeted TTGE in determining diversity of gut microbiota during acute diarrhoea and convalescence. *Journal of Health, Population and Nutrition*.

[8] Solano-Aguilar G., Fernandez K. P., Ets H. (2013). Characterization of fecal microbiota of children with diarrhea in 2 locations in Colombia. *Journal of Pediatric Gastroenterology and Nutrition*.

[9] Vandeputte D., Falony G., Vieira-Silva S., Tito R. Y., Joossens M., Raes J. (2016). Stool consistency is strongly associated with gut microbiota richness and composition, enterotypes and bacterial growth rates. *Gut*.

[10] Falony G., Joossens M., Vieira-Silva S. (2016). Population-level analysis of gut microbiome variation. *Science*.

[11] Pop M., Walker A. W., Paulson J. (2014). Diarrhea in young children from low-income countries leads to large-scale alterations in intestinal microbiota composition. *Genome Biology*.

[12] Ma C., Wu X., Nawaz M. (2011). Molecular characterization of fecal microbiota in patients with viral diarrhea. *Current Microbiology*.

[13] Cox G. J., Matsui S. M., Lo R. S. (1994). Etiology and outcome of diarrhea after marrow transplantation: a prospective study. *Gastroenterology*.

[14] Huang W., Bush A., Chao N. J., Chen B. J., Sung A. D. (2017). Syngeneic fecal microbiota transplant effectively attenuated graft-versus-host disease after bone marrow transplantation in mice. *Blood*.

[15] Jenq R. R., Taur Y., Devlin S. M. (2015). Intestinal blautia is associated with reduced death from graft-versus-host disease. *Biology of Blood and Marrow Transplantation*.

[16] Taur Y., Xavier J. B., Lipuma L. (2012). Intestinal domination and the risk of bacteremia in patients undergoing allogeneic hematopoietic stem cell transplantation. *Clinical Infectious Diseases*.

[17] Shono Y., Docampo M. D., Peled J. U. (2016). Increased GVHD-related mortality with broad-spectrum antibiotic use after allogeneic hematopoietic stem cell transplantation in human patients and mice. *Science Translational Medicine*.

[18] Holler E., Butzhammer P., Schmid K. (2014). Metagenomic analysis of the stool microbiome in patients receiving allogeneic stem cell transplantation: loss of diversity is associated with use of systemic antibiotics and more pronounced in gastrointestinal graft-versus-host disease. *Biology of Blood and Marrow Transplantation*.

[19] Khoruts A., Sadowsky M. J. (2016). Understanding the mechanisms of faecal microbiota transplantation. *Nature Reviews Gastroenterology & Hepatology*.

[20] Ma Y., Liu J., Rhodes C., Nie Y., Zhang F. (2017). Ethical issues in fecal microbiota transplantation in practice. *American Journal of Bioethics*.

[21] Carmo J., Marques S., Chapim I. (2015). Leaping forward in the treatment of Clostridium difficile infection: update in 2015. *GE Portuguese Journal of Gastroenterology*.

[22] Kakihana K., Fujioka Y., Suda W. (2016). Fecal microbiota transplantation for patients with steroid-resistant acute graft-versus-host disease of the gut. *Blood*.

[23] Spindelboeck W., Schulz E., Uhl B. (2017). Repeated fecal microbiota transplantations attenuate diarrhea and lead to sustained changes in the fecal microbiota in acute, refractory gastrointestinal graft-versus-host-disease. *Haematologica*.

[24] Lee C. H., Steiner T., Petrof E. O. (2016). Frozen vs fresh fecal microbiota transplantation and clinical resolution of diarrhea in patients with recurrent clostridium difficile infection: a randomized clinical trial. *JAMA*.

[25] Faith D. P., Baker A. M. (2007). Phylogenetic diversity (PD) and biodiversity conservation: some bioinformatics challenges. *Evolutionary Bioinformatics Online*.

[26] Andermann T., Peled J. U., Ho C. (2018). Microbiome-host interactions in hematopoietic stem cell transplant recipients. *Biology of Blood and Marrow Transplantation*.

[27] Graf D., Cagno R. D., Fåk F. (2015). Contribution of diet to the composition of the human gut microbiota. *Microbial Ecology in Health & Disease*.

[28] Andreyev J., Ross P., Donnellan C. (2014). Guidance on the management of diarrhoea during cancer chemotherapy. *Lancet Oncology*.

[29] Hamilton M. J., Weingarden A. R., Sadowsky M. J., Khoruts A. (2012). Standardized frozen preparation for transplantation of fecal microbiota for recurrent Clostridium difficile infection. *American Journal of Gastroenterology*.

[30] Kelly C. P. (2012). Current strategies for management of initial Clostridium difficile infection. *Journal of Hospital Medicine*.

[31] Bilinski J., Grzesiowski P., Sorensen N. (2017). Fecal microbiota transplantation in patients with blood disorders inhibits gut colonization with antibiotic-resistant bacteria: results of a prospective, single-center study. *Clinical Infectious Diseases*.

[32] DeFilipp Z., Peled J. U., Li S. (2018). Third-party fecal microbiota transplantation following allo-HCT reconstitutes microbiome diversity. *Blood Advances*.

[33] Kelly C. R., Ihunnah C., Fischer M. (2014). Fecal microbiota transplant for treatment of Clostridium difficile infection in immunocompromised patients. *American Journal of Gastroenterology*.

[34] Odamaki T., Kato K., Sugahara H. (2016). Age-related changes in gut microbiota composition from newborn to centenarian: a cross-sectional study. *BMC Microbiology*.

[35] Xu H. B., Jiang R. H., Sheng H. B. (2017). Meta-analysis of the effects of Bifidobacterium preparations for the prevention and treatment of pediatric antibiotic-associated diarrhea in China. *Complementary Therapies in Medicine*.

[36] Madan J. C., Hoen A. G., Lundgren S. N. (2016). Association of cesarean delivery and formula supplementation with the intestinal microbiome of 6-week-old infants. *JAMA Pediatrics*.

[37] Kelly C. R., Kahn S., Kashyap P. (2015). Update on fecal microbiota transplantation 2015: indications, methodologies, mechanisms, and outlook. *Gastroenterology*.

[38] Halfvarson J., Brislawn C. J., Lamendella R. (2017). Dynamics of the human gut microbiome in inflammatory bowel disease. *Nature Microbiology*.

[39] Chang J. Y., Antonopoulos D. A., Kalra A. (2008). Decreased diversity of the fecal Microbiome in recurrent clostridium difficile-associated diarrhea. *Journal of Infectious Diseases*.

[40] Stark P. L., Lee A. (1982). The microbial ecology of the large bowel of breast-fed and formula-fed infants during the first year of life. *Journal of Medical Microbiology*.

[41] Palmer C., Bik E. M., DiGiulio D. B., Relman D. A., Brown P. O. (2007). Development of the human infant intestinal microbiota. *PLoS Biology*.

[42] Seth A., Yan F., Polk D. B., Rao R. K. (2008). Probiotics ameliorate the hydrogen peroxide-induced epithelial barrier disruption by a PKC- and MAP kinase-dependent mechanism. *American Journal of Physiology-Gastrointestinal and Liver Physiology*.

[43] Resta-Lenert S., Barrett K. E. (2003). Live probiotics protect intestinal epithelial cells from the effects of infection with enteroinvasive *Escherichia coli* (EIEC). *Gut*.

[44] Resta-Lenert S., Barrett K. E. (2006). Probiotics and commensals reverse TNF-alpha- and IFN-gamma-induced dysfunction in human intestinal epithelial cells. *Gastroenterology*.

[45] Thompson A. L., Monteagudo-Mera A., Cadenas M. B., Lampl M. L., Azcarate-Peril M. A. (2015). Milk- and solid-feeding practices and daycare attendance are associated with differences in bacterial diversity, predominant communities, and metabolic and immune function of the infant gut microbiome. *Frontiers in Cellular and Infection Microbiology*.

[46] Backhed F., Roswall J., Peng Y. (2015). Dynamics and stabilization of the human gut microbiome during the first year of life. *Cell Host & Microbe*.

[47] Tannock G. W., Lawley B., Munro K. (2013). Comparison of the compositions of the stool microbiotas of infants fed goat milk formula, cow milk-based formula, or breast milk. *Applied and Environmental Microbiology*.

[48] Koenig J. E., Spor A., Scalfone N. (2011). Succession of microbial consortia in the developing infant gut microbiome. *Proceedings of the National Academy of Sciences*.

[49] Fallani M., Amarri S., Uusijarvi A. (2011). Determinants of the human infant intestinal microbiota after the introduction of first complementary foods in infant samples from five European centres. *Microbiology*.

[50] Vanderhoof J. A. (1999). Lactobacillus GG in the prevention of antibiotic-associated diarrhea in children. *Journal of Pediatrics*.

[51] Osterlund P., Ruotsalainen T., Korpela R. (2007). Lactobacillus supplementation for diarrhoea related to chemotherapy of colorectal cancer: a randomised study. *British Journal of Cancer*.

